# Prevalence of *Helicobacter pylori* Infection in Children From Households With Members' History of Successful *H. pylori* Eradication

**DOI:** 10.1002/jgh3.70453

**Published:** 2026-07-30

**Authors:** Trang Dinh Linh, Van Hoang Thi Hai, Thuong Nguyen Thu, Uyen Vu Thi Thu, Trang Tran Thi Thu, Anh Le Quynh, Long Hoang Bao, Long Dao Van, Hang Dao Viet

**Affiliations:** ^1^ School of Preventive Medicine and Public Health Hanoi Medical University Hanoi Vietnam; ^2^ Institute of Gastroenterology and Hepatology Hanoi Vietnam; ^3^ Hanoi University of Pharmacy Hanoi Vietnam; ^4^ College of Health Sciences VinUniversity Hanoi Vietnam; ^5^ Faculty of Internal Medicine Hanoi Medical University Hanoi Vietnam; ^6^ Endoscopy Center Hanoi Medical University Hospital Hanoi Vietnam

**Keywords:** children, *Helicobacter pylori*, household clustering, intrafamilial transmission, Vietnam

## Abstract

**Aims:**

This study investigated the prevalence of *Helicobacter pylori* infection and associated factors in children from households with members' history of successful *H. pylori* eradication.

**Methods:**

A cross‐sectional study was conducted from February to December 2024 in Hanoi, Vietnam. Eligible households were those having lived together for at least 12 months, including at least one child under 16 years old and at least one member with successful *H. pylori* eradication. *H. pylori* infection status among family members was assessed using stool antigen testing. Data on household demographic characteristics, childcare practices, and infection status of family members were collected. Multivariable logistic regression was used to identify factors associated with infection in children.

**Results:**

A total of 193 households with 877 members were included, including 338 children under 16 years old. The prevalence of *H. pylori* infection was 59% overall and 53% among children. Children living with both infected parents had a higher infection prevalence than those with only one infected parent (58% vs. 38.2%, *p* < 0.001), with a similar trend observed among infected siblings. After adjustment, parental infection status remained the strongest predictor of infection (aOR = 3.18; 95% CI: 1.83–5.52), while larger household size was inversely associated (aOR = 0.74; 95% CI: 0.58–0.93).

**Conclusions:**

Children's infection risk increased according to the infection status of parents and siblings, while a higher number of family members was associated with a lower likelihood of infection, although this finding should be interpreted with caution, supporting the importance of household‐level transmission and family‐based prevention strategies in high‐prevalence settings.

## 1 Introduction

*Helicobacter pylori* ( *H. pylori* ) infection remains one of the most common chronic bacterial infections worldwide, affecting nearly half of the world's population [1, 2]. Infection with *H. pylori* is typically acquired during childhood, and if left untreated, it often persists throughout life [3].

*Helicobacter pylori* has been recognized as a risk factor for chronic gastritis, peptic ulcer disease, and gastric cancer, substantially contributing to the global burden of morbidity and mortality from gastrointestinal diseases [4]. Although most children infected with *H. pylori* remain asymptomatic, a proportion may develop gastritis and peptic ulcer disease and progress to gastric adenocarcinoma or mucosa‐associated lymphoid tissue lymphoma later in their adulthood [5]. Despite the declining prevalence in many high‐income countries, *H. pylori* infection remains highly prevalent in low and middle‐income settings, including many countries in Southeast Asia [1].

Evidence from previous studies has indicated that *H. pylori* transmission occurs predominantly within households, particularly through close interpersonal contact and shared living environments [6, 7]. Parents, siblings, and other cohabiting family members have been identified as important sources of infection for children [8, 9]. Previous studies have reported higher infection rates among children whose parents or siblings were infected, supporting the role of intrafamilial transmission [10, 11]. However, the strength of these associations varies across populations, and the relative contribution of different household members, such as parents, grandparents, and siblings, remains insufficiently characterized, particularly in multi‐generational living households.

In addition, although several studies have examined *H. pylori* prevalence in children or adults separately, fewer investigations have systematically assessed the infection clustering of *Helicobacter pylori* in family members within the same household. Even fewer studies have explored gradients of infection risk in children according to the number and relationship of infected household members, while simultaneously accounting for household size and socioeconomic characteristics. This gap is especially relevant in settings where extended family living arrangements are common and where household‐level prevention strategies, such as family‐based screening or simultaneous treatment of *H. pylori* , are being increasingly considered.

Vietnam is a country with a high prevalence of *H. pylori* infection and a substantial burden of related gastrointestinal diseases [8, 12]. Many families have multiple generations living together, potentially facilitating the intrafamilial transmission [10]. In Vietnam, co‐residence with children is the most prevalent living arrangement among older persons, with 61.3% residing with at least one child, reflecting frequent and prolonged intergenerational contact within households [13]. Despite growing evidence supporting intrafamilial transmission of *H. pylori* , important knowledge gaps remain regarding how the infection occurs in households with a history of infection, or even with successful eradication of a family member. In particular, limited data are available on how children's infection risk varies according to the infection status of cohabiting family members after eradication has been achieved in at least one household member. So, among children living in households where at least one family member had previously been infected with and successfully eradicated *H. pylori* , how does the children's *H. pylori* infection rate vary according to the infection status of other household members? Understanding these patterns is essential for evaluating the effectiveness of current treatment strategies and for informing family‐based prevention approaches in high‐prevalence settings.

Therefore, this study aimed to determine the prevalence of *H. pylori* infection among family members and to examine the association between household infection status and *H. pylori* infection in children, using data from households undergoing *H. pylori* testing. Specifically, we assessed the status of *H. pylori* infection among children according to the infection status of parents, grandparents, and siblings, and identified factors independently associated with *H. pylori* infection in children.

## 2 Methods

### 2.1 Study Design and Population

This study is part of a longitudinal study that followed up family members of households of a patient who had been diagnosed with *H. pylori* infection and successfully treated. In this study, we focused on child members and their baseline infection status. We recruited households that had members living together for at least 12 months, had at least one individual with complete *H. pylori* eradication therapy, included at least one family member under 16 years old, and consented to *H. pylori* stool antigen testing. Individuals receiving antibiotics or proton pump inhibitors (PPIs) were advised to discontinue these medications at least 4 weeks before *H. pylori* testing. Those experiencing acute gastrointestinal infections accompanied by diarrhea, or the presence of blood or mucus in the stool, as well as women who were menstruating, were also advised to postpone *H. pylori* testing until all symptoms had resolved and stool consistency returned to a normal form, as described by the Bristol Stool Form Scale [14]. The target population of this study was households in which at least one member had a history of *H. pylori* infection and successful eradication, representing a higher‐risk group for intrafamilial transmission rather than the general population. Recruitment occurred at Hoang Long General Clinic and Institute of Gastroenterology and Hepatology, Hanoi, Vietnam, between February and December 2024.

### 2.2 Sample Size and Sampling Methods

Convenience sampling was applied. This approach was adopted to efficiently identify households meeting the inclusion criteria, particularly those with a documented history of *H. pylori* infection and successful eradication. The minimum required sample size was calculated using the formula for estimating a population proportion:

$$n=z_{1-\frac{\alpha }{2}}^{2}\frac{p\left(1-p\right)}{{∆}^{2}}$$

Based on a study by Chen et al., which reported a prevalence of *H. pylori* infection in children of 22.2% [15], and using a margin of error of ±5% with 95% confidence, the minimum sample size was calculated as 266 children.

### 2.3 Study Procedures

A representative household member (aged ≥ 18 years) who had completed *H. pylori* eradication therapy at Hoang Long Clinic and the Institute of Gastroenterology and Hepatology and met the inclusion and exclusion criteria was invited to participate in the study. After providing informed consent, information was collected from the household representative using a standardized form, including the number of household members and their characteristics (age, sex, and relationship to the child: grandparent, parent, or sibling), occupation, monthly income of the representative, and child caregiving practices, including school meals and the practice of pre‐chewing food for children. Pre‐chewing of food is a traditional feeding practice, defined as food being chewed by the mother or other relatives before being given to the child, along with the oral microbiota [16].

From each household member, test results were collected using stool antigen tests for *H. pylori*. Household members were provided with Quick Chaser *H. pylori* test kits (Mizuho Medy Co. Ltd., Japan) along with testing instructions (flyers and videos) [17, 18]. At enrollment, the household representative provided a list of participating members, including their name, age, sex, and relationship to the representative. Participants performed the test according to the instructions and sent photographs of the results to the investigators. Each submitted image included the labeled test cassette and participant identification information for verification.

### 2.4 *H. pylori* Testing

**FIGURE 1:**
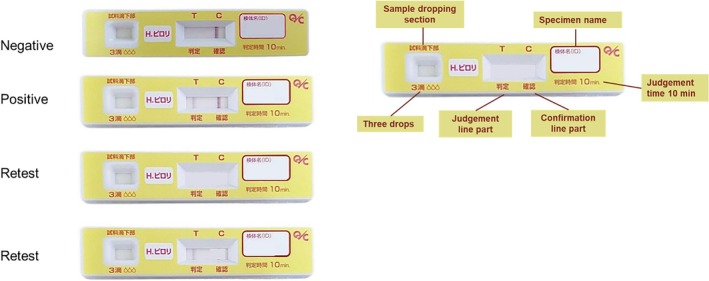
Interpretation of the *H. pylori* antigen test. The test cassette includes a control line (C) and a test line (T). A positive result is indicated by the presence of both the C and T lines; a negative result is indicated by the presence of the C line only; and an invalid result is indicated by the absence of the C line, regardless of the T line.

This is an immunochromatographic assay based on monoclonal antibodies, manufactured by Mizuho Medy Co. Ltd., Japan. The reported reaction time is 3–10 min (10 min for a negative result) (Figure 1). According to the manufacturer, compared with immunochromatographic testing, QCP has a sensitivity of 95.3%, specificity of 94.7%, and accuracy of 95.0%. When validated against ELISA (*Enzyme‐Linked Immunosorbent Assay*), the sensitivity is 100%, specificity 92.6%, and accuracy 95.7% [18]. In addition, QCP has been reported to have a sensitivity and specificity of 87.5% and 100%, when validated against the ^13^C‐urea breath test (UBT) or rapid urease test (RUT) [17].

### 2.5 Statistical Analysis

Descriptive statistics for categorical variables are presented as frequencies and percentages, while continuous variables are presented as means (standard deviations) and medians (interquartile ranges). Family infection status (parents, grandparents, and siblings) was categorized into three groups: none infected, either one infected, and all members infected (where applicable). Differences in infection proportions across these categories were evaluated using the chi‐square test. To identify factors associated with *H. pylori* infection, we first employed univariable logistic regression for all candidate variables. Factors showing potential associations or clinical relevance were then included in a multivariable logistic regression model. Specifically, a bidirectional stepwise selection procedure was applied to refine the model, with a p‐entry criterion of < 0.05 and a *p*‐removal criterion of > 0.10. Categorical variables with more than two levels, such as parents' monthly income and occupations, were converted into dummy variables for inclusion in the model. A *p*‐value < 0.05 was considered statistically significant. All analyses were done using Stata 17 (StataCorp LLC, College Station, TX, USA).

### 2.6 Ethical Consideration

This study used baseline data from the project titled “Factors associated with reinfection rate and effectiveness of family‐based management interventions in *Helicobacter pylori* successfully eradicated patients,” approved by the Ethics Committee of Hanoi Medical University (IRB No. 1221/GCN‐HMUIRB dated January 09th, 2024). All data were anonymized and securely stored to ensure participant confidentiality and were used solely for scientific research purposes. Written informed consent was obtained from a household representative on behalf of all family members, including children under 18. *H. pylori* stool antigen testing was provided at no cost to participants, as expenses were covered by the research.

## 3 Results

**FIGURE 2:**
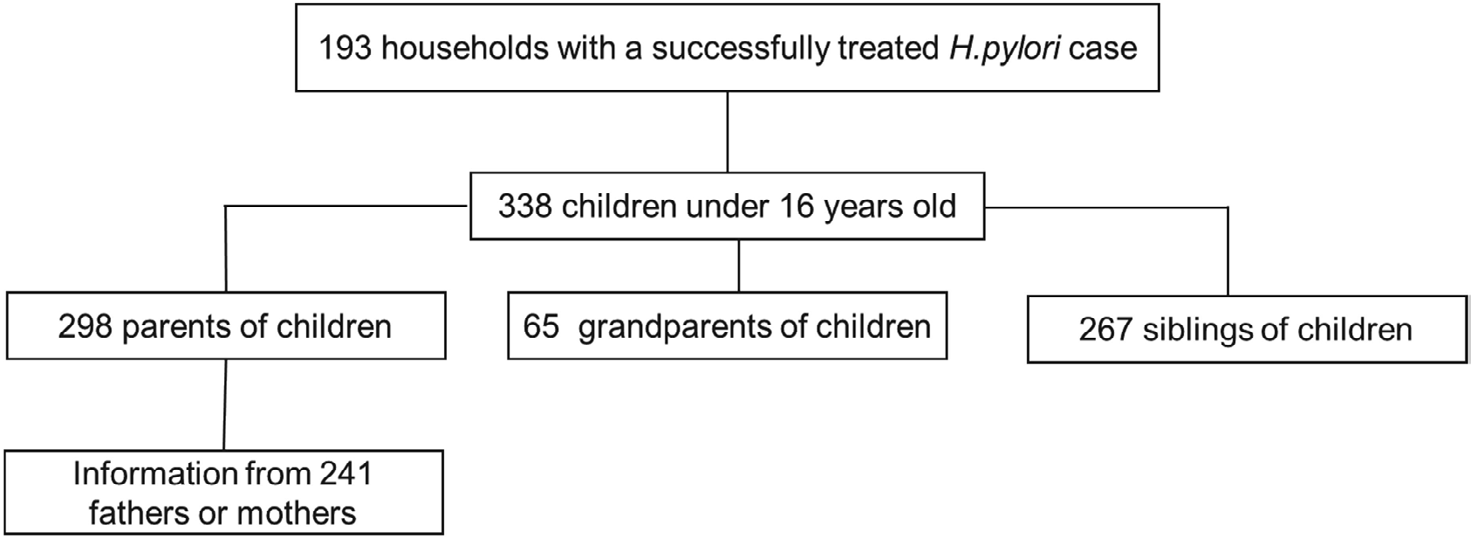
Study flow diagram.

Data was collected from 193 households, including 193 index patients with successful *Helicobacter pylori* eradication and 684 family members who underwent *H. pylori* testing using the QCP method. In total, 877 members were included in the analysis, of whom 338 were children under 16 years of age (Figure 2). Individuals who had successfully undergone eradication therapy at the time of study enrollment were classified as positive due to past infection (diagnosed by urease test and 13C‐urea breath test). The overall positivity rate in the study population was 59% (*n* = 517), and 53% (*n* = 179) of children under 16 years were *H. pylori* positive.

Based on relationships with the child, positivity rates were 40.5% in grandfathers, 66.9% in grandmothers, 66.1% in fathers, 67.3% in mothers, and 59.6% in siblings. Among parents, 43.2% were white‐collar workers, 41.9% were freelancers, and 14.9% were manual laborers. Most parents reported a monthly income exceeding 10 million VND (61.9%), while 30.1% earned between 5 and 10 million VND and 8.0% earned less than 5 million VND.

The median age of children was 9 years (interquartile range [IQR]: 5–12), and 44.7% were male. Overall, 80.3% of children had lunch with other children at school, and 53.9% of those children were *H. pylori* positive. Approximately 15 children (4.5%) had a history of receiving pre‐chewed food from their parents (frequency: rarely to often), and the difference between groups was statistically significant. Most households used treated water and had toilets located inside the households, and children from households that used untreated water showed a higher proportion of *H. pylori* positivity (Table 1).

**TABLE 1:** Prevalence of *H. pylori* positivity in children by family distribution.

Characteristics	Total (*n* = 338)	*H. pylori* positive (*n* = 179)	*H. pylori* negative (*n* = 159)
Sex, *n*%			
Male	187 (55.3)	95 (53.1)	92 (57.9)
Female	151 (44.7)	67 (42.1)	84 (46.9)
Age, median [IQR]	9 [5–12]	9 [6–12]	9 [4–13]
Age group, *n* (%)			
1–5 years	89 (26.3)	41 (22.9)	48 (30.2)
6–11 years	138 (40.8)	80 (44.7)	58 (36.5)
12–16 years	111 (32.8)	58 (32.4)	53 (33.3)
Having lunch with other children at school, *n* (%)	269 (80.3)	145 (81.5)	124 (79.0)
Pre‐chewing of food, *n* (%)*	15 (4.5)	3 (1.7)	12 (7.6)
Number of family members, median [IQR]*	4 [3–5]	4 [3–5]	4 [3–5]
Water source, *n* (%)			
Processed	269 (80.5)	147 (54.7)	122 (45.4)
Unprocessed	65 (19.5)	31 (47.7)	34 (52.3)
Toilet location*			
Inside the household	285 (84.6)	158 (55.4)	127 (44.6)
Outside the household	52 (15.4)	21 (40.4)	31 (59.6)

Abbreviation: IQR, interquartile range.

* *p* < 0.05 between the two groups.

**FIGURE 3:**
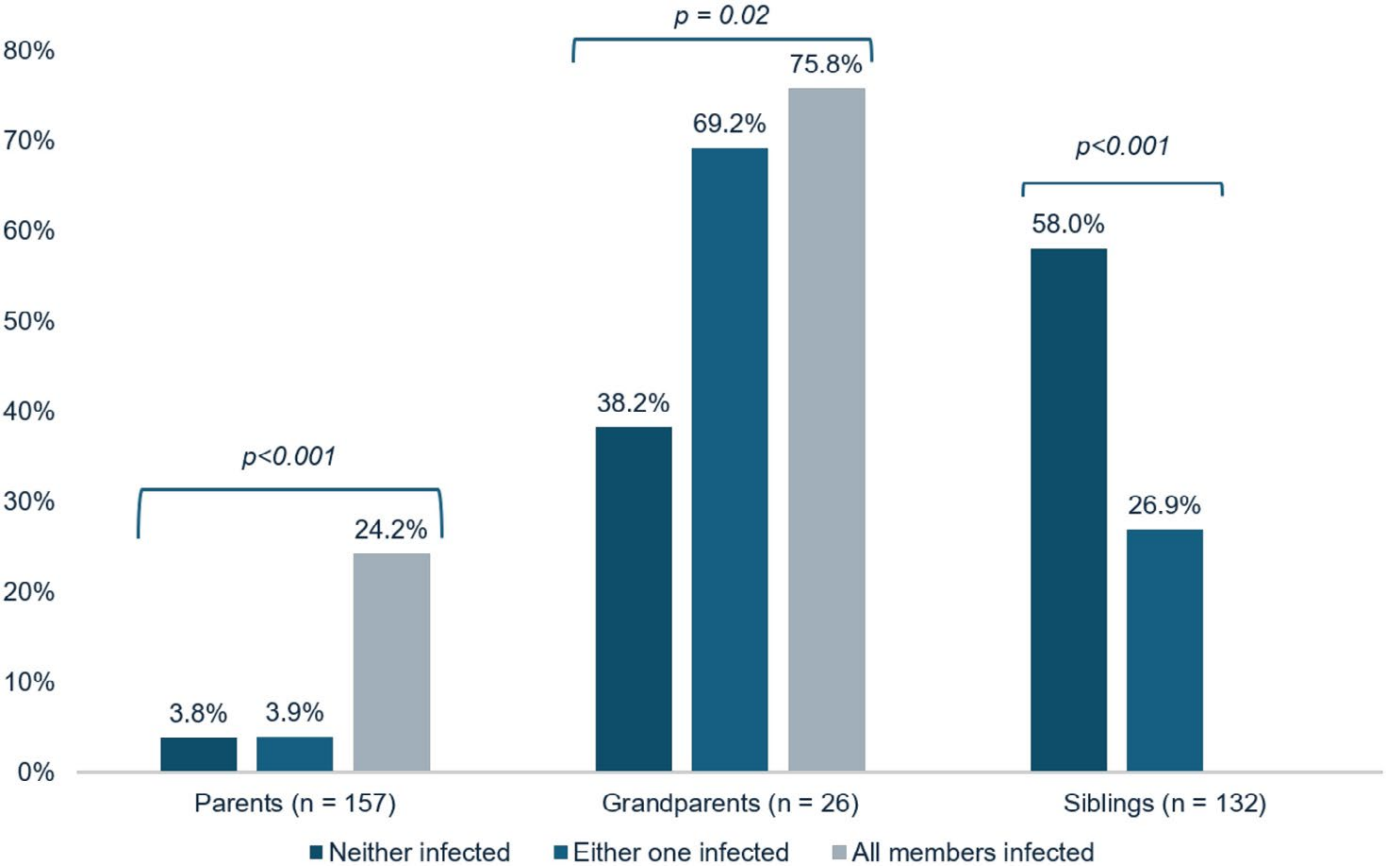
Association between parental *H. pylori* infection and child infection status.

Among the 179 infected children, the likelihood of infection increased with the number of infected household members. In the parental group (*n* = 157), 38.2% of children were infected when either the father or mother was infected, and this proportion rose to 58.0% when both parents were infected (*p* < 0.001) (Figure 3). In the univariable logistic regression model, compared with children whose parents were both uninfected, those with one infected parent had a 3.5‐fold higher odds of infection (OR = 3.5, 95% CI: 1.35–9.09, *p* = 0.01), which increased to 9.6‐fold when both parents were infected (OR = 9.6, 95% CI: 3.68–25.15, *p* < 0.001). A similar pattern was observed among children living with infected siblings, where 75.8% of children were infected when at least one sibling was infected (OR = 6.02, 95% CI: 3.51–10.31, *p* < 0.001).

Among the 241 children with complete parental information, the prevalence of infection in children was 54.8%. In the initial multivariable logistic regression model, all demographic characteristics, sanitation conditions (water source and toilet location), lifestyle habits (having lunch with other children at school, pre‐chewing of food), and parental infection status were included in the analysis. Children with infected parents had higher odds of infection compared with those whose parents were uninfected (aOR = 2.91, *p* < 0.001). Through the bidirectional stepwise selection process, parental infection status remained the strongest predictor (aOR = 3.18, *p* < 0.001). In addition, the number of family members was identified as a protective factor (aOR = 0.79, *p* = 0.02). Other factors, including child age, sex, parental occupation, water source, and toilet location, were not significantly associated with infection in the final model (Table 2).

**TABLE 2:** Multivariable logistic regression between children's *H. pylori* infection status and some related factors (*n* = 241).

Characteristics	Full model (Initial)		Final model (Stepwise)	
	aOR (95% CI)	*p*	aOR (95% CI)	*p*
Age	1.01 (0.94–1.08)	0.88	—	—
Sex				
Female	Reference		—	—
Male	0.98 (0.55–1.73)	0.94		
Number of family members	0.79 (0.60–1.05)	0.11	0.74 (0.58–0.93)	**0.02**
Having lunch with other children at school	1.01 (0.48–2.16)	0.31	—	—
Infection status of parents				
Either father or mother is infected	Reference		Reference	
Both parents infected	2.91 (1.63–5.18)	**< 0.001**	3.18 (1.83–5.52)	**< 0.001**
Pre‐chewing of food	0.30 (0.03–3.06)	0.31	—	—
Parents' monthly income				
Under 5 million	Reference		Reference	
From 5 to 10 million	0.38 (0.11–1.25)	0.11	0.60 (0.33–1.09)	0.09
Over 10 million	0.57 (0.17–1.92)	0.36	—	—
Parents' occupations				
Manual workers	Reference		—	—
Freelancers	1.25 (0.45–3.41)	0.67		
White‐collar workers	1.10 (0.59–2.04)	0.76		
Water source				
Unprocessed	Reference		—	—
Processed	1.25 (0.50–3.17)	0.64		
Toilet location				
Inside the household	Reference		—	—
Outside the household	0.74 (0.30–1.80)	0.5		

*Note:* Stepwise selection with *p*‐entry < 0.05 and *p*‐removal > 0.10. The bold values in Table 2 indicate *p* < 0.05.

Abbreviations: aOR, adjusted odds ratio; CI, confidence interval.

## 4 Discussion

In this study, we examined the distribution of *H. pylori* infection within households and identified factors associated with infection among the child population. Our findings demonstrated a high prevalence of *H. pylori* infection in children and evidence of infection clustering within families (53%). Children's infection risk increased progressively according to the infection status of parents, grandparents, and siblings, supporting the role of intrafamilial transmission in this setting. Recent family‐based studies in Asia consistently demonstrate household clustering and parent‐to‐child transmission. Children's infection was significantly associated with the infection status of household members, including parents and siblings. In the study by Huang et al., maternal infection was associated with a 2.58‐fold higher risk of child infection (OR = 2.58, 95% CI: 1.37–4.87) [10]. Similarly, paternal infection increased the odds of child infection by 1.68 times (*p* = 0.02), while maternal infection was associated with 1.70 times higher odds (*p* = 0.017) in a study by Zhou et al. [11].

The observed gradient in children's *H. pylori* infection according to parental infection status in our study was consistent with previous family‐based studies, which have shown higher infection rates among children living with infected parents, especially when both parents are *H. pylori* positive (68.9%) [5, 19]. This pattern was also comparable to a community‐based study conducted in Vietnamese multi‐generational households, which reported that children's infection prevalence was 58.5% when both parents were infected, 43.6% when either parent was infected, and 28.0% when neither parent was infected. These findings support the role of parental infection status as an important marker of intrafamilial transmission risk [9]. Our study demonstrated that children living with both parents infected with *H. pylori* had 3.18‐fold higher odds of infection compared with those living with only one infected parent (OR = 3.18; 95% CI 1.83–5.52; *p* < 0.001). The substantially higher prevalence among children whose parents were infected, compared with those whose parents were uninfected, suggests cumulative exposure through close and sustained contact. Similar patterns observed with infected siblings further support the importance of horizontal transmission within households, especially among children who share living spaces and daily activities. A meta‐analysis of 3181 records conducted by Yuan et al. reported a global prevalence of *H. pylori* infection among children of 32.3%. The study further demonstrated that living in households with infected siblings or an infected mother was associated with approximately threefold higher risks of infection (siblings: OR = 3.33, 95% CI 1.53–7.26; mother: OR = 3.31, 95% CI 2.21–4.98). In addition, having a greater number of siblings or children (OR = 1.84, 95% CI 1.44–2.36) and sharing rooms (OR = 1.98, 95% CI 1.49–2.40) were also significantly associated with increased infection risk [5]. Another study by Huang et al. similarly reported that maternal, paternal, or sibling *H. pylori* infections were associated with an increased risk of infection in children, with maternal infection conferring 2.58‐fold higher risk (OR = 2.58; 95% CI 1.37–4.87) [10]. Together, these findings reinforce the notion that household members represent a key reservoir for *H. pylori* transmission to children.

When examining factors associated with infection status in children, we considered demographic characteristics, household socioeconomic conditions, hygiene factors, and childcare practices. After adjustment, parental infection status remained the strongest factor associated with H. pylori infection. Our study findings are consistent with those of Zhou et al., a large national family‐based study that demonstrated a clear gradient in children's infection prevalence according to parental infection status (neither parent infected, one parent infected, or both parents infected), which provided strong support for the notion that parental infection status represents the most influential factor in household transmission [11]. Evidence from our study also suggested that sociodemographic factors such as age, sex, and socioeconomic status may become non‐significant after multivariable adjustment, although this contrasts with findings from previous studies in which these factors remained significant. For instance, Chen et al. reported in their multivariable analysis that higher household income remained a significant protective factor, indicating that socioeconomic status may retain an independent association with infection risk in certain settings [15]. In contrast to findings in which age was not independently associated with infection risk, Le et al. reported that older children (≥ 11 years) had a significantly higher risk of infection, as indicated by adjusted odds ratios, suggesting that the effect of age may vary depending on the study settings and sampling procedures [20].

The number of family members was inversely associated with *H. pylori* infection in children (OR = 0.74, *p* < 0.05), suggesting a potential protective effect. In Vietnam, most households are multi‐generational, with older adults often living with their adult children and spouses [21]. This finding indicates that a larger household size does not necessarily reflect a higher level of close contact. Similarly, households with three or more generations living together were associated with a lower risk of infection in a previous study (OR = 0.79) [11]. Transmission in multigenerational living environments may be less driven by static residential density and more by the frequency and intensity of close interpersonal interactions during shared daily activities, such as caregiving, food sharing, and communal eating.

An unexpected finding in this study was the lower prevalence of *H. pylori* infection among children who reported receiving pre‐chewed food. However, this result should be interpreted with caution. First, the number of children exposed to this practice was very small (*n* = 15), limiting statistical power and increasing the likelihood of random errors. Second, the association was not statistically significant in the multivariable model, suggesting that it may be explained by residual confounding or sampling variability. Existing evidence does not support a protective role of pre‐chewing or premastication. On the contrary, premastication has been proposed as a potential pathway for oral‐oral transmission of *H. pylori* and other pathogens, as it involves direct transfer of saliva from caregiver to child [22]. Therefore, the observed inverse pattern in this study was unlikely to reflect a true protective effect, and no causal interpretation should be made. Further studies with a larger sample size and more detailed assessment of feeding practices are needed to clarify the role of pre‐chewing food in *H. pylori* transmission.

From a public health perspective, these findings have important implications. The strong clustering of *H. pylori* infection within households suggests that individual‐based testing and treatment strategies may be insufficient in high‐prevalence settings. Family‐based screening and simultaneous treatment approaches, particularly targeting households with infected parents or siblings, may be more effective in reducing reinfection of *H. pylori* in persons who have successfully undergone eradication treatment and interrupting the intra‐familial transmission. Such approaches may be especially relevant in countries like Vietnam, where *H. pylori* prevalence remains high and multi‐generational living households are common.

This study acknowledges several limitations. Its cross‐sectional design precluded causal inference and does not allow determination of the direction or timing of transmission of *H. pylori* within households. In addition, the convenience sampling method from clinical settings might introduce selection bias, as households recruited from specialized gastroenterology clinics might differ from the general population in terms of health‐seeking behaviors, disease awareness, or underlying risk factors. Information on certain behavioral factors, such as specific feeding practices or hygiene behaviors, was limited. Moreover, the household representatives invited to participate in the study could be grandparents or older siblings of children under 16 years of age, so we were unable to collect information on the parents' occupation or income. Not all household members were tested in every family, as some declined to undergo testing or use medicine in exclusion criteria, which may have led to an underestimation of the prevalence of *H. pylori* infection. Non‐participation of some household members might have introduced exposure misclassification and biased the estimation of household clustering, although the extent and impact of this missing data could not be formally assessed. Furthermore, because not all household members underwent testing in every family, the substantial amount of missing data prevented us from incorporating the infection status of grandparents and siblings into the multivariable regression models. Nevertheless, the relatively large sample size and the inclusion of multiple household members in our study strengthened the validity of the observed associations.

## 5 Conclusion

In conclusion, our findings provided evidence of pronounced intrafamilial clustering of *H. pylori* infection in Vietnamese households and highlight a graded increase in children's infection risk according to the infection status of parents and siblings. Interestingly, a higher number of family members was associated with a lower likelihood of infection in children. Although this finding should be interpreted with caution, it suggests that households dynamics may be more complex than conventional assumptions regarding transmission risk. These results underscore the importance of considering household structure and family‐based approaches in *H. pylori* prevention and control strategies, particularly in high‐prevalence and multi‐generational settings.

## Funding

The authors have nothing to report.

## Conflicts of Interest

The authors declare no conflicts of interest.

## Data Availability

The data that support the findings of this study are available from the corresponding author upon reasonable request.
